# How Health Communication via Tik Tok Makes a Difference: A Content Analysis of Tik Tok Accounts Run by Chinese Provincial Health Committees

**DOI:** 10.3390/ijerph17010192

**Published:** 2019-12-27

**Authors:** Chengyan Zhu, Xiaolin Xu, Wei Zhang, Jianmin Chen, Richard Evans

**Affiliations:** 1 College of Public Administration, Huazhong University of Science and Technology, Wuhan 430030, China; 2016512018@hust.edu.cn (C.Z.); xiaolin@hust.edu.cn (X.X.); 2 School of Medicine and Health Management, Huazhong University of Science and Technology, Wuhan 430030, China; m201975543@hust.edu.cn; 3 College of Engineering, Design and Physical Sciences, Brunel University London, Kingston Lane, Uxbridge, Middlesex UB8 3PH, UK; richard.evans@brunel.ac.uk

**Keywords:** micro-video, Provincial Health Committee, healthcare, Tik Tok, social media, China

## Abstract

During the last two decades, social media has immersed itself into all facets of our personal and professional lives. The healthcare sector is no exception, with public health departments now capitalizing on the benefits that social media offers when delivering healthcare education and communication with citizens. Provincial Health Committees (PHCs) in China have begun to adopt the micro-video sharing platform, Tik Tok, to engage with local residents and communicate health-related information. This study investigates the status quo of official Tik Tok accounts managed by PHCs in mainland China. In total, 31 PHC accounts were analyzed during August 2019, while the top 100 most liked micro-videos were examined using content analysis. Coding included three major aspects: Quantified Impact, Video Content, and Video Form. 45.2% (n = 14) of PHCs had official Tik Tok accounts. A limited number of accounts (n = 2) were yet to upload a micro-video, while most (n = 9) had uploaded their first micro-video during 2019. For the top 100 most liked micro-videos, a sharp difference was observed in terms of number of Likes, Comments and Reposts. Videos containing cartoons or documentary-style content were most frequently watched by citizens. Similarly, content that promoted professional health or provided knowledge of diseases was frequently viewed. Content containing original music, formal mandarin language, subtitles, and which lasted less than 60 s, were most frequently followed. It is considered a missed opportunity that most PHCs struggle to take advantage of the Tik Tok platform, especially given its growing popularity and daily increase in account creation.

## 1. Introduction

### 1.1. Social Media Use by Healthcare Departments

Since the inception of social media in the late 1990s, it has had an unprecedented influence on our personal and professional lives, impacting the way we communicate, stay connected, and share information. By July 2019, the number of worldwide active social media users reached 3.534 billion, with a penetration rate of 46% [1]. Undoubtedly, the popularity and ease in adoption of social media has changed the ways in which public services are delivered and communicated. An increasing number of government agencies have realized the importance of actively participating on social media for citizen engagement, relationship building, and citizen compliance [2,3,4,5,6]. One recently introduced social media platform is Tik Tok, a micro-video sharing platform that allows users to create short videos, lasting from several seconds to several minutes, and then share it with the wider Tik Tok community. Founded in 2017, it is the fastest growing social media application in the world, topping the chart for ‘Most Downloaded’ in the USA in 2018, and now being available in over 150 countries. It is claimed that Tik Tok has more than 500 million active users with more than 1 billion downloads [7]. By contrast with other social media platforms, Tik Tok is characterized by short micro-videos with simple-to-use editing and music-inclusion functions [8,9]. The application provides a non-complicated user interface for creating videos, with users being able to embed their preferred music choices and special effects into their recorded video, easily [9].

In the healthcare sector, the adoption of social media is not new [10,11]. Social media has been widely adopted by patients, care-givers, and healthcare professionals, with numerous studies reporting its usefulness in patient empowerment, health promotion, patient–physician relationship building, public health surveillance, and quality improvement [12,13,14,15,16,17]. On the contrary, other studies have focused on revealing the dark side of social media in healthcare; for example, examining how unverified content leads to the sharing of misleading information [18], patients becoming overconfident in their own medical decision making [19,20], and privacy violation [18,21]. Scholars have also noted the level of diffusion of social media among healthcare departments [22], observing differences in content shared and patient interactions [23]. Since healthcare departments concern the quality in medical care delivered to citizens, their adoption of social media is often cautious and can lag behind other public-facing sectors.

### 1.2. The Use of Tik Tok by Government Agencies in China

In October 2016, the State Council of China established the Healthy China 2030 Strategy, aimed at promoting healthier lifestyles, improving health literacy and mental health. In the process of developing the strategy, health communication was viewed as critical for providing access to public health information, education and health literacy improvement. In July 2019, the Healthy China Promotion Committee of the State Council strengthened the importance of establishing and improving the “Two databases and one mechanism” for public health education. The two databases refer to the database of national and provincial experts in public health education and the database of national public health education resources. One mechanism refers to a holistic media perspective on public health education and communications, denoting that health communication via integrated media forms has garnered national attention.

In China, social media usage is the most popular online activity, with most citizens now focusing their attention on creating and watching micro-videos. According to the 44th report of Internet development in China, produced by CNNIC (China Internet Network Information Center), the number of mobile internet users has reached 817 million, with the number of micro-video users now exceeding 648 million, showing a penetration rate of 75.8% by June 2019 [24]. The popularity of Tik Tok has also attracted local government attention. In China, government organizations have begun to use Tik Tok to engage with local citizens, enabling them to clarify public concerns and keep citizens informed [25,26]. Tik Tok has also penetrated local health authorities in China, although at a much-reduced rate compared with the large-scale adoption witnessed by local governments. This study explores this emerging phenomenon in China where local health committees are utilizing the micro-video sharing platform to provide health information to the general public. It examines how Chinese citizens are participating on the platform, analyzing the top 100 most liked videos from PHCs to determine the most common features and communication strategies favored by the general public. The results of our study will enrich the understanding of social media use by PHCs, drawing comparisons against previous social media types and providing guidance for the future operation of Tik Tok accounts.

## 2. Materials and Method

### 2.1. Data Collection

In this study, 31 provincial level health committees in mainland China were examined. Data was captured on 20 August 2019. All PHCs were classified based on their provincial economic development capacity and geographic location, including the Western, Central and Eastern regions. To begin, we searched the Tik Tok platform for the official accounts of all PHCs in China by using the official name of the health committee and abbreviations of their name. If no result was found, we located the official website of the committee and checked if there were any links to their official Tik Tok account [27]. To ensure that all Tik Tok accounts were official, we analyzed only those that were verified accounts on the Tik Tok platform, represented by a blue check mark (verified badge).

### 2.2. Analysis of Tik Tok Accounts

A Microsoft Excel worksheet was constructed to store the data extracted from the official Tik Tok accounts. The extraction was completed by one person, the third author. The data was cross-sectional in nature and included: (1) established time of the account i.e., date since the first uploaded Tik Tok video, (2) number of videos posted, (3) number of likes received, (4) number of video reposts, and (5) number of comments received.

To further analyze the content of uploaded micro-videos, content analysis of the top 100 most liked micro-videos among all PHCs was conducted. Content analysis is widely used when analyzing video-based health communication [28,29,30,31], e.g., obesity videos [32] and e-cigarettes [33]. However, many studies that have adopted content analysis mainly examined videos lasting from several minutes to hours. Their coding usually includes content types, subtitles, valence, and specifically designed codes based on topics being studied. In our study, the coding scheme for micro-videos produced by healthcare departments on Tik Tok is still immature; therefore, we followed the codes of common practice for video-based health communication and piloted the coding scheme with an initial analysis of 20% (20/100) of the selected micro-videos. We then made amendments as further videos were processed [16,34]. The final coding scheme consisted of three dimensions: Quantified Impact, Video Content, and Video Form. Each dimension had several sub-dimensions.

The Quantified Impact does not intend to measure the effect of watching a micro-video on an individual’s health literacy or their health-related behavioral change. Instead, we record the index observable via Tik Tok, for example how many people watched it, how many people liked it after watching it, and how many people shared it. We did not aggregate the index into one due to the difficulty in compiling a formula to evaluate their impact. Video Content concerns the video type, major themes, embedded emotions, and the characters involved in the video. Specifically, video theme refers to the topic referred to in the video, including disease knowledge, daily diet, health professionals’ image promotion, healthcare, and health reform. Disease knowledge features specific information on types and causes of disease e.g., the cause of hypertension and treatment available to the patient. Daily diet provides insight into developing healthy eating habits. Videos on health professionals image promotion center on stories of good doctors, nurses, hospital managers, administrators and others employed in healthcare field, which highlight the individuals positive image rather than specific medical knowledge e.g., a cardiovascular surgeon saved a patient experiencing a sudden heart attack on a high-speed train. Healthcare information refers to healthy lifestyles, including diet and exercise activities. Health reform views the topic of health from a wider perspective, the policy dimension, referring to e.g., policies surrounding family doctors. Finally, emotion refers to the major emotion presented within the video, rather than the emotion triggered by watching the video. We created four sub-dimensions based on a previous classification on the emotions involved in Tik Tok, and made this fit the health communication setting [26]. Video Form concerns the style, language, and special techniques used. Specifically, the background music relates to how the music is selected, with original music being defined as the music being made by the uploader themselves, rather than them selecting a piece of music from the Tik Tok music library. 

**Figure 1:**
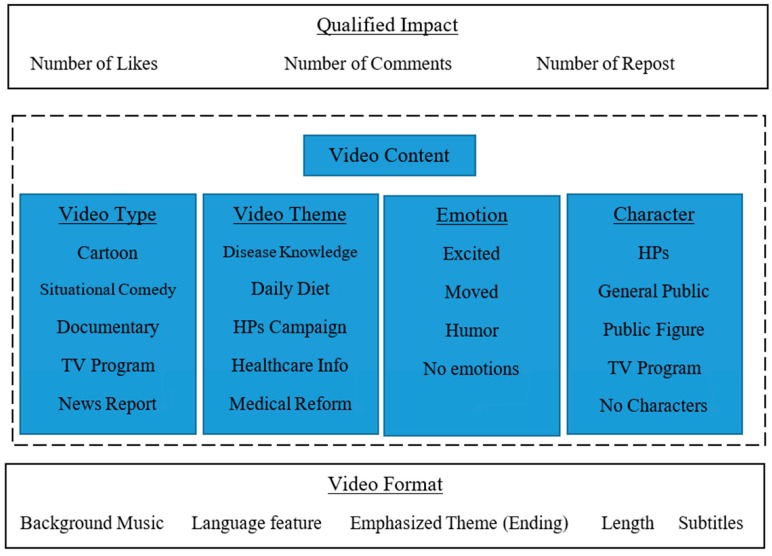
The coding framework for the top 100 most liked micro-videos run by Provincial Health Committees (PHCs).

In total, 12 codes were merged. Specifically, the Quantified Impact dimension had three sub-dimensions: Number of Likes, Comments and Reposts. The video content dimension had four sub-dimensions: Video type, Theme, Emotion, and Characters. The video form dimension included five sub-dimensions: Background Music, Language Feature, Emphasized Theme in the ending, Length of video, and Subtitles. The final coding scheme is presented in Figure 1 and Table 1. Two graduate students studying Health Informatics were trained before coding the micro-videos separately, with their inter-reliability rate being 0.94 [6,35], deeming the coding scheme effective.

## 3. Results

Of the 31 provincial level health committees observed, 14 (45.2%) had their own official Tik Tok account, including 6 located in the Central region of China, and 4 in the East and West, respectively. Among them, the health committees in Tianjin and Shanghai had not uploaded any videos, although they had created official accounts. Health committees in Shanxi were the first to release a micro-video on Tik Tok in 2018, while many (64.3%, 9/14) had uploaded their first micro-video in 2019. The total number of followers for all health committees in China was 197,980. The total number of videos uploaded was 962, while the total number of Likes received had reached 1.054 million. 

In terms of the 100 most liked micro-videos, none were posted by health committees in Henan, Hunan, Hainan, Guizhou, Inner Mongolia or the NingXia autonomous regions. Instead, health committees from Shanxi, Jilin, Jiangxi, Hubei, Guangdong, and Sichuan provinces had the most popular micro-videos. Table 2 provides further details of the most liked videos.

### 3.1. Distribution of Quantified Impact Dimension

Quantified Impact is defined as the measurement of an official Tik Tok account’s influence on the public engagement via Tik Tok. It differs in the top 100 micro-videos in terms of the total number of likes, comments and reposts. Specifically, the total number of likes of the top 100 micro-videos had reached 775,256, ranging from 667 to 177,000. The total number of comments received were 13,579 with zero, being the least, to 6815. Among them, the number of micro-videos that received comments exceeding 1000 was only two, while most micro-videos received less than 100 comments. The total number of reposts amounted to 59,568, with zero being the least and 20,000 being the most. Eleven micro-videos had been reposted at least 1000 times. Table 3 presents further details.

### 3.2. Distribution of Video Content Dimension

Video content was divided into four sub-dimensions. In terms of video type, videos involving cartoons took the lead with 27% (27/100), followed by those which involved a documentary (25%, 25/100) or except from TV program (22%, 22/100). For video theme, the topic of health professionals ranked the highest, with 38 out of 100 videos displaying this topic. Videos presenting knowledge about diseases was second, with slightly fewer micro-videos, while the theme of daily diets and health reform was viewed the least number of times with 7% (7/100) and 3% (3/100), respectively. In terms of the emotion involved in the video, over half (58%, 58/100) demonstrated no specific feelings. The feeling of being moved was felt in 19 out 100 micro-videos, followed by the feeling of sense of humor (17%, 17/100), and being excited (6%, 6/100). In terms of characters, 55 out of 100 videos featured healthcare professionals, 12% (12/100) focused on public figures, and 11% (11/100) on the general public, with patients included. The remaining 22% presented no characters.

### 3.3. Distribution of Video Form Dimension

The format of uploaded micro-videos was divided into five sub-dimensions. For background music used, it was split into three categories: no background music, default music selected from Tik Tok, and original music made by the uploader. 82 out of 100 micro-videos were accompanied by various types of music. Among them, 63.4% (52/82) included original music, produced by the PHC. For the language features, we looked at the local dialects used. Only 3% (3/100) of the micro-videos used local dialects. Mandarin was used in the majority micro-videos for the avoidance of language barriers caused by regional dialects. However, the use of dialects may attract locals in some way.

In terms of length of time of the micro-video, an overwhelming number of videos (98, 98/100) were presented within 60 s. Among them, 11 lasted 0–15 s, 16 from 15–30 s, 28 from 30–45 s, and 43 from 45–60 s. Regarding subtitles, 73 out of 100 showed subtitles. For emphasized theme in the ending scene, only 20% (20/100) of the micro-videos had included this.

## 4. Discussion

### 4.1. Principal Findings

This study has provided an overview of the official Tik Tok accounts managed by provincial health committees across mainland China. According to the results, 45.2% (14/31) of the provincial health committees in mainland China have created a Tik Tok account, at the time of data collection. 12 of them had already uploaded their first micro-video, except for those in Tianjin and Shanghai, while many (n = 9) PHCs had uploaded their first micro-video in 2019. It is interesting to note that the adoption of Tik Tok by health departments in provinces with economic prosperity (e.g., Tianjin and Shanghai) lagged behind others. This may be due to the overall health literacy rates in these regions, and effective health education through earlier-adopted social media channels (e.g., Weibo and Wechat). In the case of provinces with economic prosperity, citizens are likely to have a higher level of health literacy, with local health departments delivering better health education. Earlier forms of social media have well-established reputations in communicating health information, which mean the marginal benefit of Tik Tok remains subtle. The diffusion patterns of Tik Tok may not simply apply to economic prosperity and openness, and other factors such as the seriousness of Tik Tok and urgency of adoption, should be taken into consideration. This is consistent with a recent study revealing the complexity of Tik Tok adoption in China, which highlighted the unique function, orientation, production procedures and communicational features compared with pre-existing social media [36]. In our study, we focused on the top 100 most popular micro-videos of all PHCs. Over half of the micro-videos were produced by the Guangdong health committee. For the Quantified Impact, although the PHCs had gained some popularity, a sharp difference was observed between the top 100 most liked micro-videos, in terms of the number of Likes, Comments and Reposts. Many accounts were still in their infancy stage. In terms of video content, many videos showed cartoons or were a documentary produced by the PHC. Themes relating to the promotion of healthy professional images and disease knowledge were most frequently seen in the observed micro-videos. In terms of video format, videos incorporating original music, using formal mandarin language, lasting less than 60 s, and using subtitles, were most commonly followed.

### 4.2. Followers Are Fundamental to Health Communication on Tik Tok

The effective communication of healthcare information via social media requires considerable followers. Our study revealed that the top 100 most liked micro-videos were mainly from six PHCs, who each had a large following. For example, the Guangdong health committee contributed 53 of the most liked micro-videos and has 130,000 followers, uploading more than 478 micro-videos with over 650,000 likes. Among all accounts, the impact of *Healthy Guangdong* has contributed to more than half of the total number of followers and Likes. At present, *Healthy Guangdong* is the most valuable Tik Tok account from the perspective of quantified impact. This could provide solid evidence that the use of social media by local health departments is often more cautious than that of their counterparts in other government agencies. Since Tik Tok adoption by PHCs is still novel in China, many may be hesitant in its use. In addition, they may have also not mastered the strategies for health communication via the emerging social media channel. Their investment in account promotion and creation of more attractive micro-videos needs strengthening. PHCs are advised to integrate the current impact of other online platforms and establish their reputation on Tik Tok. To achieve this, PHCs can use a social media management tool e.g., Hootsuite, to manage cross-posting of videos across social media channels and schedule content, enabling more consistent posting of content at times which maximize engagement. Furthermore, an introduction or weblink to the official Tik Tok account could be displayed on each PHC official website, Weibo or Wechat account to migrate audiences and build a larger community on Tik Tok.

To attract followers in future, PHCs could create micro-videos related to or engaging with popular topics i.e., reflecting on subjects that concern the general public. By using these as headlines or cover pages, it is likely to gain more public attention. Although popular topics may not be health-related, the micro-video can still be created with the popular topic in-mind. However, the determinants for continued attention still largely depend on the quality of each micro-video. Tik Tok users may be more engaged with a micro-video connected with a popular topic at first but will often become disinterested if the video is not entertaining. To overcome this, PHCs could engage with regionally-admired influencers from e.g., sport or film in China to grow the number of followers and increase public engagement. Since the majority of Tik Tok users are drawn to the platform by entertaining content, better efforts are encouraged to design health-education oriented videos that incorporate comedic elements. PHCs should further consider the creation of docuseries surrounding interesting topics, creating messaging strategies that feature consistent characters e.g., “A Day in the Life of Doctor Z”. Meanwhile, they could make greater use of external resources related to public health education, collaborating more frequently with local medical institutes, media and health non-governmental organizations (NGOs), to amplify their influence. For example, in the popular micro-video titled ‘*I Am A Doctor Not A God*’, a rap is created by the Sichuan health committee and Luzhou Renmin Hospital to celebrate the Chinese doctor’s day. The rap was adapted from a recent blockbuster film ‘*Dying to Survive’* or ‘*I Am Not A God Of Medicine*’ and has been popularized since it’s upload to Tik Tok.

### 4.3. Easily Understood Content and Light Format Are Critical

Our study revealed that Tik Tok users prefer the presentation and format of micro-videos to be correlated to their understanding of difficult medical terms or jargons. With regards to content type, animated cartoons and documentary were seen to be popular for content relating to demonstration and for creating a sense of being ‘in the moment’, to stimulate shared emotion, compared to oral presentations and plain text which were preferred for the sharing of disease knowledge and information on specific drugs. This is consistent with the notion that the narrative story-telling approach has been increasingly adopted to disseminate health-related content in health communication [37]. Meanwhile, the promotion of a health professional’s image and disease knowledge were major themes currently viewed. This could be attributed to citizens wanting to get to know their healthcare professionals to obtain different perspectives. For example, a situation-based comedy on overloaded workloads of doctors at New Year Eve brings the public closer to doctors’ daily lives, while a micro-video on hypertension management attracts the attention of patients and their care-takers. In addition, the dominant role in micro-videos were that of healthcare professionals. This may be due to medicine being a highly specialized topic where years of training and practice are required before developing informed knowledge. Also, health professionals, including physicians, doctors, and public servants are often easily trusted by the public when sharing health information. The strategy of using public figures for health communication may also be worth applauding. For example, actress Yang Zi and actor Zhang Yishan, favored by young citizens in China, played roles in a scene representing and discussing depression prevention issues. 

In terms of format used, a light style in terms of background music, language, length, and subtitles is preferred by content viewers. Although these peripheral factors are not deciding issues when determining video quality, the format could serve as a motivating factor for increasing viewing time [38]. For the creation of health-related micro-videos, PHCs are expected to follow regular patterns in health communication. For example, videos which contain complicated music or too many visual effects can easily distract the audience. The use of vivid cartoons can demonstrate disease knowledge, however, this format could undermine key health information; if one can only remember the cartoon figures, but nothing about the health knowledge, the communication is ineffective. The key health education message should be essential for all micro-video formats. Further efforts are also required to facilitate training and management of Tik Tok account operations for PHCs. To manage Tik Tok accounts more efficiently, PHCs are expected to learn from their peers, as well as other government departments, and allocate more resources to support the development of content and operation of the account. 

### 4.4. Audience-Centered Interactions Are Encouraged

Compared to the total number of Likes and Reposts per micro-video, the total number of Comments was far lower. This may indicate that the public treat health communication via Tik Tok as one-way communication rather than two-way communication. The Tik Tok accounts currently managed by PHCs had few interactions with their audiences; video co-creation can be a means for extending communication between creators and recipients. This is a commonly mentioned problem in previous literature when new information technologies are introduced into public sectors [10,39]. Tik Tok is a valuable co-creation community where the health department can promote healthy lifestyles and impart health-related knowledge, while the users can also generate health related information to impact others, marketers and policy makers. Further interactions require the creators to activate a pro-interaction environment, engage audiences in the content-creation and promotion process, and thus realize the multi-dimensional exchanges and interactions between audiences, service platform and micro-videos. 

It could be speculated that micro-videos uploaded by PHCs do not provide enough features for the public to participate in conversation. Meanwhile, in the case of China, where video-based social media has long been regarded as an entertainment platform for amusement, health or science related communication has largely been neglected; a nudge approach by the health department may gradually change the perception hindering effective health communication. In future, PHCs are expected to partner with the Tik Tok platform to improve the image of Tik Tok as a health communication channel. In addition, the audience profile of PHCs is worthy of attention. The analysis of audience characters, i.e., age and locality, will contribute to making targeted health communication strategies more applicable to citizens. If audiences are dominated by young citizens, the most common youth health-related problems should be addressed in the content. Additionally, the video created should consider their preference, daily routine and lifestyle. To initiate conversations, PHCs should find common health concerns, and encourage public participation in the process of creating the micro-video. For example, PHCs could create a micro-video based on popular user discussions, using buzzwords to gain public attention, thus engaging the public in health-related communication.

### 4.5. Limitations

This study has certain limitations. First, it was a descriptive study exploring PHC use of the Tik Tok platform, a newly introduced micro-video sharing platform. We selected the top 100 most liked micro-videos produced by PHCs, however, number of Likes may not sufficiently measure the popularity of each video, as lots of accounts had been newly created. We did not consider the number of Reposts and Comments. Longer duration is also required to see more dynamics of this new social media platform. Meanwhile, although content analysis is widely used in video analysis, it may still have limitations when demonstrating the special features of micro-videos studied for short duration. Future research can enlarge the study sample, using machine learning or other automatic techniques e.g., using transcriptions, shoot analysis, sentiment analysis or narrative analysis to reveal a more comprehensive picture of Tik Tok accounts run by PHCs.

Second, the reasons for social media adoption and their usage strategies were understudied. As our study centered on the use of Tik Tok accounts, it is possible that other important characteristics may be neglected. For example, the specific conditions of each PHC including administration, level of resources, and public engagement. Meanwhile, our study did not examine the health-related changes resulted from Tik Tok, and additional comparison of the health effect via different social media channels could be of great value to understand the social media use in healthcare.

Third, in our research, we did not investigate the operators of Tik Tok accounts, regarding their selection of content, abiding by the rules, and internal rewards for managing the account. This could lead to variations in Tik Tok adoption. Future study could employ qualitative methods to reveal the ‘behind the scenes’ story and compare their strategic use across different social media platforms. In addition, the exploration of the recipients of health communication videos via Tik Tok is also worth further attention. For instance, qualitative work that looks into their attitude of watching Tik Tok run by PHCs and their expectations on the content.

## 5. Conclusions

Less than half of all PHCs in mainland China have established an official Tik Tok account. The value of the Guangdong PHC account is the highest in terms of total number of followers, Likes and Reposts. Content analysis of the top 100 most liked health-related micro-videos revealed that many PHCs are new to operating Tik Tok accounts and struggle to leverage this emerging social media platform. Animated cartoons and documentary-style content, featuring healthcare professionals’ and disease knowledge are popular among viewers of health-related micro-videos. More than half of the selected micro-videos focused on health professionals with no specific emotions involved. In addition, original music and subtitles are also easily observed from selected micro-videos. Considering that Tik Tok is a micro-video based social media platform, patient-centered interactions are highly desired for PHCs to create more diverse content with engaging strategies. With many social media users, especially younger generations, now preferring to receive information via video content over written content, PHCs should use Tik Tok to grow engagement levels with citizens, creating content that is unique and personable, and which extends the core values of the PHC. Tik Tok should be viewed as an official means for communicating health information with citizens and not purely an entertainment channel; instead, it should be become an integral part of PHCs social media ecosystems, allowing agencies to interact with citizens on a more personal level, extending communication away from merely written form. 

## Figures and Tables

**Table 1:** The coding scheme for video analysis.

Index	Explanation
Number of Likes	Total number of Likes by 20 August 2019
Number of Comments	Total number of Comments received by 20 August 2019
Number of Reposts	Total number of Reposts by 20 August 2019
Video Type	Refers to the different types of health communication, divided into five categories, including: cartoon, documentary, situation comedy, excerpt from TV program, excerpt from news report.
Video Theme	Refers to the major topic involved in the micro-video, encompassing disease knowledge, daily diet, health professionals’ image promotion, healthcare info, and health reforms.
Emotion	Refers to the major emotion involved; classified as excited, moved, humor or no specific emotion.
Character	Refers to the character playing the leading role or being shown most during the micro-video; divided into health professionals, public figures and general public (with patients included).
Background Music	Refers to the background music used, including no music, music selected from the Tik Tok music library, and original music.
Language Feature	Refers to the language used, including mandarin, and other local dialects.
Emphasized theme (Ending)	Refers to the technique of re-emphasizing the theme at the end of the micro-video.
Length	Refers to the length of the micro-video.
Subtitles	Refers to the technique of using subtitles to display the words spoken in the micro-video as written text.

**Table 2:** Basic information of Tik Tok accounts run by PHCs.

Province	Region	First Time Video Uploaded	Number of Followers	Number of Uploaded Videos	Number of Updates	Total Number of Likes	Number of Top 100 most liked Micro-Videos
Tianjin	East	Never	26	0	0	0	0
Shanghai	East	Never	4	0	0	0	0
Shanxi	Central	21 June 2018	5145	133	133	31,000	11
Jilin	Central	19 April 2019	13,000	74	75	127,000	14
Jiangxi	Central	12 May 2019	614	19	19	2808	1
Henan	Central	4 April 2019	190	5	5	367	0
Hubei	Central	5 May 2019	8566	121	121	45,000	5
Hunan	Central	10 July 2019	58	5	5	140	0
Guangdong	East	4 September 2018	132,000	478	527	651,000	53
Hainan	East	30 November 2018	274	9	9	580	0
Sichuan	West	23 January 2019	38,000	97	105	196,000	16
Guizhou	West	19 August 2019	54	12	12	51	0
Inner Mongolia	West	25 April 2019	38	6	6	13	0
Ning Xia	West	16 August 2019	11	3	3	27	0
Total			197,980	962	1,015	1,053,986	100

**Table 3:** The distribution of the Quantified Impact dimension.

Quantified Impact	Min	Max	Median	Sum
Number of Likes	667	177,000	7,752.56	775,256
Number of Comments	0	6815	135.97	13,597
Number of Reposts	0	20,000	595.68	59,568

## References

[1] Wearesocial Global Social Media Users Pass 3.5 Billion. http://wearesocial.cn/blog/2019/07/22/global-social-media-users-pass-3-5-billion/.

[2] Agostino D., Arnaboldi M. (2016). A Measurement Framework for Assessing the Contribution of Social Media to Public Engagement: An empirical analysis on Facebook. Public Manag. Rev..

[3] Hong H. (2013). Government websites and social media’s influence on government-public relationships. Public Relat. Rev..

[4] Im T., Cho W., Porumbescu G., Park J. (2012). Internet, trust in government, and citizen compliance. J. Public Adm. Res. Theory.

[5] Song C., Lee J. (2016). Citizens’ Use of Social Media in Government, Perceived Transparency, and Trust in Government. Public Perform. Manag. Rev..

[6] Zhang W., Xu X., Zhang H., Chen Q. (2016). Online participation chaos: A case study of Chinese government-initiated e-polity square. Int. J. Public Adm..

[7] Wearesocial Digital 2019 Q2 Global Digital Statshot. http://wearesocial.cn/blog/2019/04/28/digital-2019-q2-global-digital-statshot/.

[8] Chen Z., He Q., Mao Z., Chung H.-M., Maharjan S. A Study on the Characteristics of Douyin Short Videos and Implications for Edge Caching. http://arxiv.org/abs/1903.12399.

[9] Yang S., Zhao Y., Ma Y. Analysis of the Reasons and Development of Short Video Application—Taking Tik Tok as an Example. Proceedings of the 2019 9th International Conference on Information and Social Science (ICISS 2019).

[10] Heldman A.B., Schindelar J., Weaver J.B. (2013). Social Media Engagement and Public Health Communication: Implications for Public Health Organizations Being Truly “Social”. Public Health Rev..

[11] Deng Z., Hong Z., Zhang W., Evans R., Chen Y. (2019). The Effect of Online Effort and Reputation of Physicians on Patients’ Choice: 3-Wave Data Analysis of China’s Good Doctor Website. J. Med. Internet Res..

[12] Ranney M.L., Genes N. (2016). Social media and healthcare quality improvement: A nascent field. BMJ Qual. Saf..

[13] Tengilimoglu D., Sarp N., Yar C.E., Bektaş M., Hidir M.N., Korkmaz E. (2017). The consumers’ social media use in choosing physicians and hospitals: The case study of the province of Izmir: Choosing Physicians and Hospitals. Int. J. Health Plan. Manag..

[14] Richter J.P., Muhlestein D.B., Wilks C.E. (2014). Social media: How hospitals use it, and opportunities for future use. J. Healthc. Manag..

[15] Zhang W., Deng Z., Evans R., Xiang F., Ye Q., Zeng R. (2018). Social Media Landscape of the Tertiary Referral Hospitals in China: Observational Descriptive Study. J. Med. Internet Res..

[16] Zhang W., Deng Z., Hong Z., Evans R., Ma J., Zhang H. (2018). Unhappy Patients Are Not Alike: Content Analysis of the Negative Comments from China’s Good Doctor Website. J. Med. Internet Res..

[17] Zhu C., Zeng R., Zhang W., Evans R., He R. (2019). Pregnancy-Related Information Seeking and Sharing in the Social Media Era Among Expectant Mothers: Qualitative Study. J. Med. Internet Res..

[18] Syed-Abdul S., Fernandez-Luque L., Jian W.-S., Li Y.-C., Crain S., Hsu M.-H., Wang Y.-C., Khandregzen D., Chuluunbaatar E., Nguyen P.A. (2013). Misleading Health-Related Information Promoted Through Video-Based Social Media: Anorexia on YouTube. J. Med. Internet Res..

[19] Smailhodzic E., Hooijsma W., Boonstra A., Langley D.J. (2016). Social media use in healthcare: A systematic review of effects on patients and on their relationship with healthcare professionals. BMC Health Serv. Res..

[20] Baron R.J., Berinsky A.J. (2019). Mistrust in Science—A Threat to the Patient–Physician Relationship. N. Engl. J. Med..

[21] Househ M., Borycki E., Kushniruk A. (2014). Empowering patients through social media: The benefits and challenges. Health Inform. J..

[22] Thackeray R., Neiger B.L., Smith A.K., Van Wagenen S.B. (2012). Adoption and use of social media among public health departments. BMC Public Health.

[23] Jha A., Lin L., Savoia E. (2016). The Use of Social Media by State Health Departments in the US: Analyzing Health Communication through Facebook. J. Community Health.

[24] China Network Internet Information Center (2019). The 44th China Statistical Report on Internet Development.

[25] Onion Lab and Caasdata A Report on the Analysis of Government Tik Tok Accounts in 2018. https://www.useit.com.cn/thread-20984-1-1.html.

[26] Chengwei W., Liang M. (2019). How do government micro-videos make a difference: A content analysis of government Tik Tok accounts. E Gov..

[27] Yang P.-C., Lee W.-C., Liu H.-Y., Shih M.-J., Chen T.-J., Chou L.-F., Hwang S.-J. (2018). Use of Facebook by Hospitals in Taiwan: A Nationwide Survey. Int. J. Environ. Res. Public Health.

[28] Frohlich D.O., Zmyslinski-Seelig A. (2012). The Presence of Social Support Messages on YouTube Videos about Inflammatory Bowel Disease and Ostomies. Health Commun..

[29] Gabarron E., Fernandez-Luque L., Armayones M., Lau A.Y. (2013). Identifying Measures Used for Assessing Quality of YouTube Videos with Patient Health Information: A Review of Current Literature. Interact. J. Med. Res..

[30] Tian Y. (2010). Organ Donation on Web 2.0: Content and Audience Analysis of Organ Donation Videos on YouTube. Health Commun..

[31] Wen N., Chia S.C., Hao X. (2015). What Do Social Media Say About Makeovers? A Content Analysis of Cosmetic Surgery Videos and Viewers’ Responses on YouTube. Health Commun..

[32] Yoo J.H., Kim J. (2012). Obesity in the New Media: A Content Analysis of Obesity Videos on YouTube. Health Commun..

[33] Paek H.-J., Kim S., Hove T., Huh J.Y. (2014). Reduced Harm or Another Gateway to Smoking? Source, Message, and Information Characteristics of E-Cigarette Videos on YouTube. J. Health Commun..

[34] Ma J., Zhang W., Harris K., Chen Q., Xu X. (2016). Dying online: Live broadcasts of Chinese emerging adult suicides and crisis response behaviors. BMC Public Health.

[35] Brennan R.L., Prediger D.J. (1981). Coefficient Kappa: Some Uses, Misuses, and Alternatives. Educ. Psychol. Meas..

[36] Liang M. (2019). Government Micro-video: Status quo, challenges and future directions. E Gov..

[37] Frank L.B., Murphy S.T., Chatterjee J.S., Moran M.B., Baezconde-Garbanati L. (2014). Telling Stories, Saving Lives: Creating Narrative Health Messages. Health Commun..

[38] Connelly B.L., Certo S.T., Ireland R.D., Reutzel C.R. (2010). Signaling Theory: A Review and Assessment. J. Manag..

[39] Hsu Y.-C., Chen T.-J., Chu F.-Y., Liu H.-Y., Chou L.-F., Hwang S.-J. (2019). Official Websites of Local Health Centers in Taiwan: A Nationwide Study. Int. J. Environ. Res. Public Health.

